# Supplementary material for: Conditional chemoconnectomics (cCCTomics) as a strategy for efficient and conditional targeting of chemical transmission
Source: eLife. 2024 Apr 30;12:RP91927. doi: 10.7554/eLife.91927 (PMC11060718; doi:10.7554/eLife.91927)
Supplement: Figure 3—source data 3. [file elife-91927-fig3-data3.zip › Figure 3 source data3 part 1.zip!/Figure 3-Source data 3-part1/1081-1-CRISPResso_Report_8zosk6yj.zip!/CRISPResso_on_1081_3/1c.Alignment_barplot.pdf]

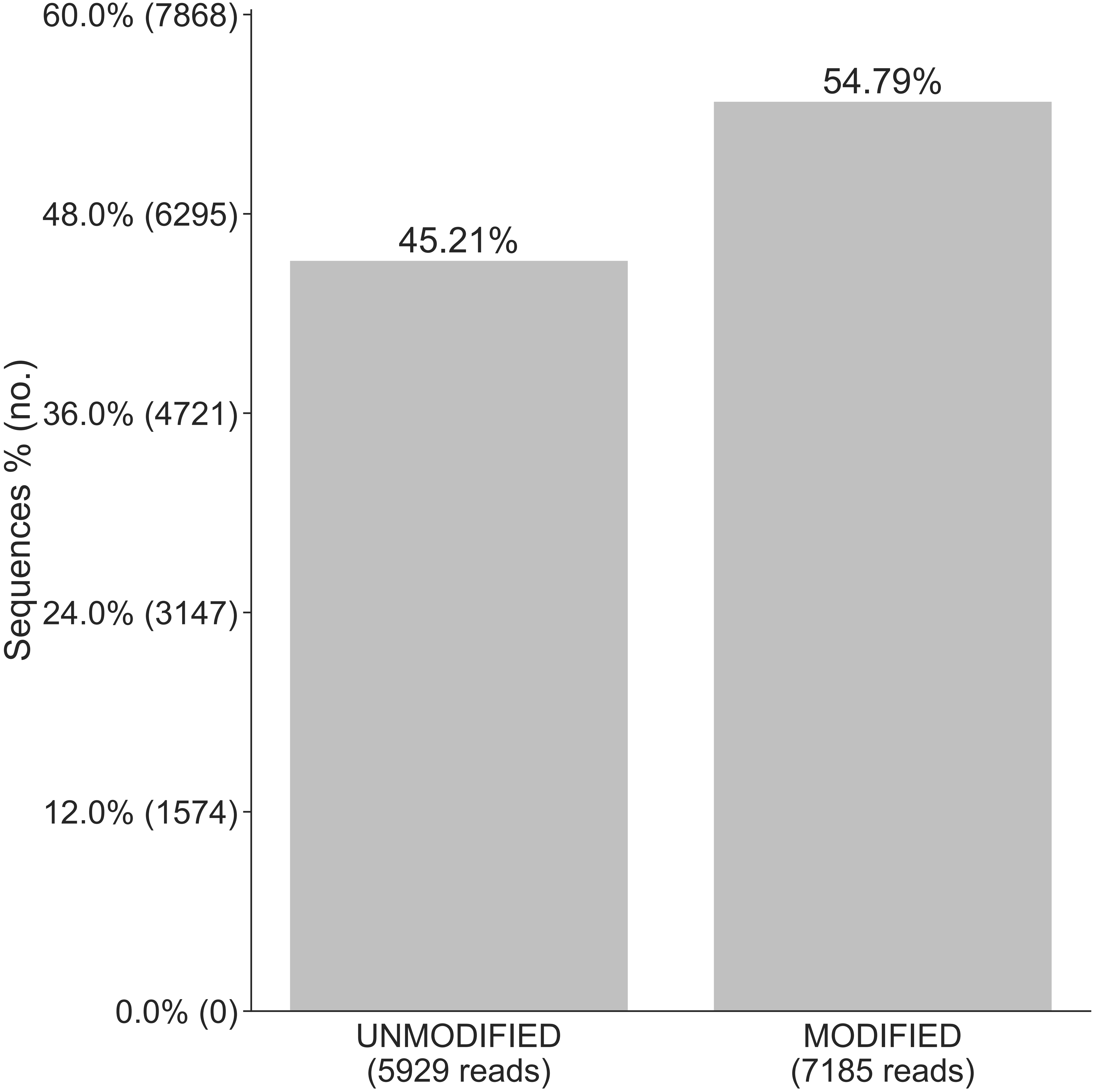

Sequences % (no.)
60.0% (7868)
48.0% (6295)
36.0% (4721)
24.0% (3147)
12.0% (1574)
0.0% (0)
45.21%
54.79%
UNMODIFIED
(5929 reads)
MODIFIED
(7185 reads)
